# Knockout of the Cardiac Transcription Factor NKX2-5 Results in Stem Cell-Derived Cardiac Cells with Typical Purkinje Cell-like Signal Transduction and Extracellular Matrix Formation

**DOI:** 10.3390/ijms241713366

**Published:** 2023-08-29

**Authors:** Paul Disse, Isabel Aymanns, Lena Mücher, Sarah Sandmann, Julian Varghese, Nadine Ritter, Nathalie Strutz-Seebohm, Guiscard Seebohm, Stefan Peischard

**Affiliations:** 1 Institute for Genetics of Heart Diseases (IfGH), Department of Cardiovascular Medicine, University Hospital Münster, D-48149 Münster, Germany; 2 Institute of Medical Informatics, University of Münster, D-48149 Münster, Germany

**Keywords:** hiPSC, cardiac conduction system, NKX2-5, connexin, collagen, CrispR-Cas9

## Abstract

The human heart controls blood flow, and therewith enables the adequate supply of oxygen and nutrients to the body. The correct function of the heart is coordinated by the interplay of different cardiac cell types. Thereby, one can distinguish between cells of the working myocardium, the pace-making cells in the sinoatrial node (SAN) and the conduction system cells in the AV-node, the His-bundle or the Purkinje fibres. Tissue-engineering approaches aim to generate hiPSC-derived cardiac tissues for disease modelling and therapeutic usage with a significant improvement in the differentiation quality of myocardium and pace-making cells. The differentiation of cells with cardiac conduction system properties is still challenging, and the produced cell mass and quality is poor. Here, we describe the generation of cardiac cells with properties of the cardiac conduction system, called conduction system-like cells (CSLC). As a primary approach, we introduced a CrispR-Cas9-directed knockout of the NKX2-5 gene in hiPSC. NKX2-5-deficient hiPSC showed altered connexin expression patterns characteristic for the cardiac conduction system with strong connexin 40 and connexin 43 expression and suppressed connexin 45 expression. Application of differentiation protocols for ventricular- or SAN-like cells could not reverse this connexin expression pattern, indicating a stable regulation by NKX2-5 on connexin expression. The contraction behaviour of the hiPSC-derived CSLCs was compared to hiPSC-derived ventricular- and SAN-like cells. We found that the contraction speed of CSLCs resembled the expected contraction rate of human conduction system cells. Overall contraction was reduced in differentiated cells derived from NKX2-5 knockout hiPSC. Comparative transcriptomic data suggest a specification of the cardiac subtype of CSLC that is distinctly different from ventricular or pacemaker-like cells with reduced myocardial gene expression and enhanced extracellular matrix formation for improved electrical insulation. In summary, knockout of NKX2-5 in hiPSC leads to enhanced differentiation of cells with cardiac conduction system features, including connexin expression and contraction behaviour.

## 1. Introduction

Heart disease is one of the leading causes of death worldwide and a burden on health systems [1,2]. The aim of being able to reverse or cure such diseases is a desire of many doctors and clinical scientists. Since the discovery of human-induced pluripotent stem cell (hiPSC) technology, scientists have been trying to use hiPSC-derived heart cells to investigate the causes of various heart diseases and also to regenerate already diseased or destroyed heart tissue [3,4,5]. However, the simple differentiation of cardiomyocytes from a hiPSC line to be used for patient treatment or disease modelling has proven to be problematic and not feasible, as hiPSC-derived cardiomyocytes do not resemble the phenotype present in a mature heart and are often limited in contractility and excitability [6]. So far, only hiPSC-based differentiations into cardiac muscle cells have progressed to the point where initial attempts at cell transplantation are being made. [7,8]. Furthermore, this approach is only suitable for the treatment of diseases of the working myocardium. However, several diseases affect other specialised regions of the heart, e.g., the sinus node (SAN) or the conduction system of the heart, for which no specific differentiation protocols are available [9,10]. The sinus node is the actual pacemaker of the heart and regulates the heart’s beating rate, while the conduction system, built up by a fibre network, transports the electrical stimuli generated by the SAN throughout the heart [11,12]. A few scientific studies have aimed to produce hiPSC-derived pacemaker-like cells in high numbers and of high quality, with an average success rate [13,14]. Approaches to differentiate specialised cells of the conduction system are even rarer and limited by the low yield of those cells [15,16,17].

In the present study, we address the lack of a valuable hiPSC differentiation method for cells of the conduction system and present a potential concept for an efficient and easy-to-use protocol for CSLC generation. The homeobox-containing transcription factor (NK2 homeobox 5; NKX2-5) is a key regulator in cardiac development that interacts with other cardiac transcription factors such as GATA4, TBX5 and MEF2A/B to activate the expression of myocardial genes [18,19]. Recent studies showed that the developing mammal heart also contains NKX2-5-deficient regions, which in the course of heart development form the SAN, and probably also the conductive fibres [20,21]. We concluded that deficiency of NKX2-5 may be a key regulator that distinguishes the forming myocardium from the cardiac conduction system during embryogenesis. Therefore, we performed a CrispR-Cas9-based knockout of the NKX2-5 gene in a standard hiPSC line and tested different differentiation protocols for cardiac subtypes to verify the formation of conduction system-like cells. Given the fact that different cardiac cell types differ in the expression levels of their gap junction-forming proteins, the connexins, we took this as a basis to examine the cell types we generated for their connexin expression pattern. As cells of the sinus node usually show a high expression of connexin 45, cells of the conduction system highly express connexin 40 and connexin 43. We distinguished the resulting cells based on their expression of different connexin proteins, which are specific for different cardiac compartments, their contraction behaviour, and their transcriptomic profile [22,23].

## 2. Results

**Figure 1:**
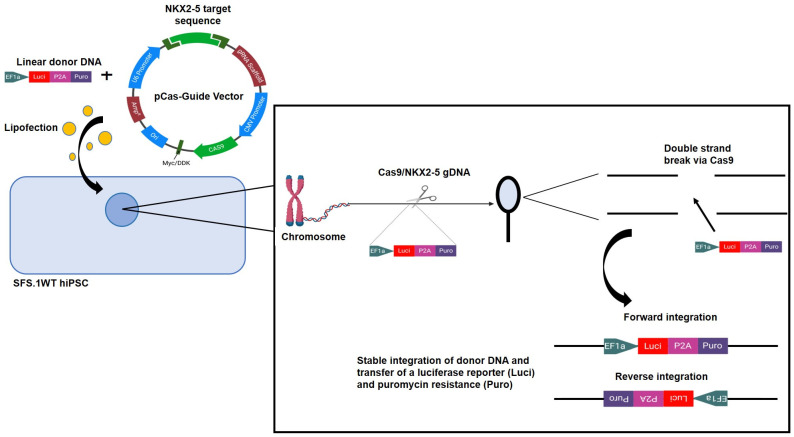
Schematic representation of the CrispR-Cas9 guided knockout of NKX2-5 in hiPSC using the pCAS-Guide vector as performed in this study. The pCAS-Guide vector and the donor DNA Luci-Puro were transfected via lipofection into SFS.1WT hiPSC. The pCAS-Guide controlled expression of Cas9 allows for the targeted knockout of the NKX2-5 gene and the integration of Luci-Puro into the accessible genome of the SFS.1WT cell.

Knockout of NKX2-5 in the hiPSC line Sendai Foreskin 1 (SFS.1WT) was performed by transfecting the pCas-Guide vector containing a NKX2-5 target sequence together with the linear donor DNA oligo Luci-P2A-Puro coding for a Luciferase reporter and Puromycine resistance, allowing for optical and chemical isolation of cell clones (Figure 1).

**Figure 2:**
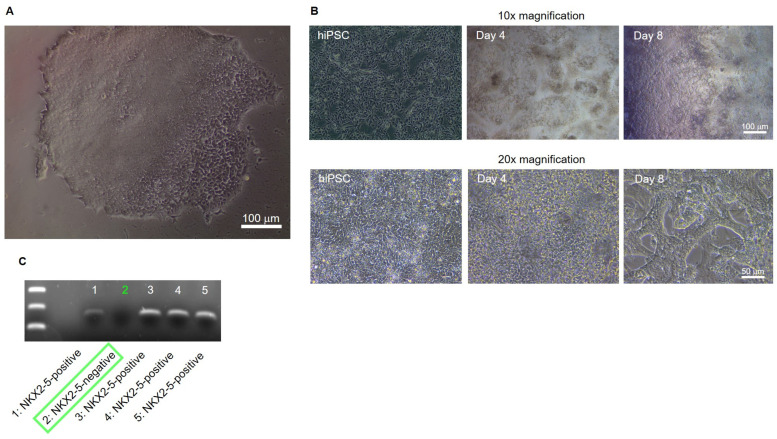
Generation of an NKX2-5KO hiPSC-line. (**A**) Exemplary hiPSC colony after 17 days of selection with Puromycin. (**B**) hiPSC culture of isolated, CrispR-Cas9-treated hiPSC colonies. The cells were differentiated towards hiPSC-derived cardiomyocytes. The pictures show the cells as hiPSC, as mesodermal/cardiac cells at day 4 of differentiation and as young cardiomyocytes at day 8 of differentiation. (**C**) Young cardiomyocytes derived from CrispR-Cas9-treated hiPSC were isolated at day 8 of differentiation and analysed for the expression of NKX2-5 via PCR. Clones 1, 3, 4, 5 show an amplified NKX2-5 fragment. Clone 2 lacks the NKX2-5 fragment at 146 bp (green square).

**Figure 3:**
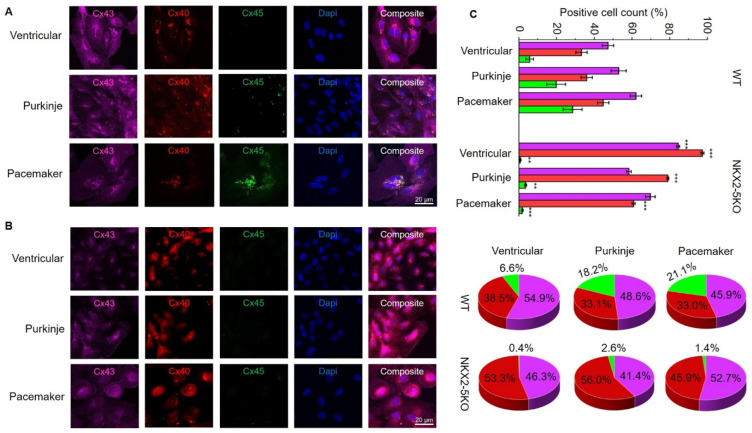
Application of cardiac subtype differentiation protocols on SFS.1WT and SFS.1-NKX2-5KO and characterisation of cardiac connexin expression patterns. (**A**) Immunofluorescence staining for connexin 43, connexin 40 and connexin 45 of SFS.1WT cells, which were differentiated with protocols for ventricular-, Purkinje- and pacemaker-like cells. (**B**) Immunofluorescence staining for connexin 43, connexin 40 and connexin 45 of SFS.1-NKX2-5KO cells, which were differentiated with protocols for ventricular-, Purkinje- and pacemaker-like cells. (**C**) Top figure; FACS analysis of SFS.1WT and SFS.1-NKX2-5KO cells, which were differentiated towards ventricular-, Purkinje- and pacemaker-like cells. The count of cells positive for connexin 43 (purple), connexin 40 (red) and connexin 45 (green) was quantified and compared (** *p* < 0.01; *** *p* < 0.001). Bottom figure; FACS analysis of SFS.1WT and SFS.1-NKX2-5KO cells, which were differentiated towards ventricular-, Purkinje- and pacemaker-like cells. The cardiac connexin expression pattern of each differentiation group was calculated and normalised to 100%. Equal differentiation conditions of SFS.1WT and SFS.1-NKX2-5KO were compared to each other.

Positively transfected clones were detected, isolated and cultured by puromycin selection (Figure 2A). After sufficient expansion of the potential NKX2-5 knockout clones, cardiac differentiation of the clones was performed until spontaneous beating occurred on day 8 of differentiation. Differentiated, young cardiomyocytes show a strong 3D structure and aggregation into contractile strands (Figure 3B). On day 8 of differentiation, cells were harvested and analysed for NKX2-5 RNA expression by PCR followed by electrophoresis. One of five clones, clone #2, was negative for NKX2-5 expression and was determined to be an NKX2-5 knockout clone (Figure 3C). Clone #2 was designated SFS.1-NKX2-5KO and used for further experiments.

For further characterisation, SFS.1WT and SFS.1-NKX2-5KO cells were differentiated into cardiac subtypes using three differentiation protocols as described in the Materials and Methods section. Cells were differentiated for 35 days and then analysed for their connexin expression pattern and cellular connexin localisation. Immunofluorescence staining of connexin 43, connexin 40 and connexin 45 showed strong expression of connexin 43 and connexin 40 in all three cardiac subtypes of SFS.1WT-hiPSC. No connexin 45 signal was observed in the ventricular differentiation protocol, and only low connexin 45 expression was detected in the Purkinje differentiation protocol. Strong expression of connexin 45 was detected in cells differentiated using the pacemaker differentiation protocol (Figure 3A). When SFS.1-NKX2-5KO cells were differentiated, connexin 43 and connexin 40 were also detected in all three differentiation types. It is worth noting that the connexin 43 signal appeared weaker compared to the SFS.1 WT differentiation types and the connexin 40 signal was stronger overall. Connexin 45 signal was abolished in all three differentiation types of SFS.1-NKX2-5KO cells (Figure 3B).

FACS sorting of differentiated SFS.1WT and SFS.1-NKX2-5KO cells for connexin 43, connexin 40 and connexin 45 signals confirmed the immunofluorescence staining observations. SFS.1WT cells differentiated with the ventricular differentiation protocol showed a high count for connexin 43-positive cells (47.31% ± 3% SEM), a high count for connexin 40-positive cells (33.20% ± 3% SEM) and a low count for connexin 45-positive cells (5.70% ± 7% SEM). When SFS.1WT cells were differentiated using the Purkinje cell differentiation protocol, connexin 43 was also found in a high count for cells (52.93% ± 4% SEM). Connexin 40 was also detected in a high count (36.00% ± 3% SEM) and connexin 45 was obtained in an increased count (19.80% ± 5% SEM) compared to SFS.1WT cells differentiated with the ventricular differentiation protocol. After differentiation of SFS.1WT with the pacemaker cell differentiation protocol, high connexin 43 (62.13% ± 3% SEM), connexin 40 (44.73% ± 3% SEM) and clear connexin 45 expression (28.50% ± 5% SEM) was observed. Ventricular differentiation of SFS.1-NKX2-5KO resulted in very high connexin 43 (84.50% ± 0.0081% SEM) and connexin 40 (97.36% ± 0.0008% SEM) counts and low connexin 45 (0.66% ± 0.0005% SEM) counts. Application of the Purkinje cell differentiation protocol to SFS.1-NKX2-5KO resulted in a high count for connexin 43 (58.41% ± 0.0120% SEM) and connexin 40 (79.06% ± 0.0050% SEM). The connexin 45 count (3.60% ± 0.0051% SEM) was far below the connexin 45 count in SFS.1WT cells treated with the Purkinje cell differentiation protocol. Application of the pacemaker differentiation protocol to SFS.1-NKX2-5KO resulted in a high count for connexin 43 (69.75% ± 0.0256% SEM) and connexin 40 (60.67% ± 0.0081% SEM). The count for connexin 45 (1.84% ± 0.0038% SEM) was greatly reduced compared to SFS.1WT cells differentiated using the pacemaker differentiation protocol (Figure 3C, top panel). Since differentiation efficiency can vary between cell batches, we calculated the percentage of the connexin expression pattern normalised to an overall signal of 100% connexin in each experiment to make the data from SFS.1WT and SFS.1-NKX2-5KO differentiations comparable (Figure 3C bottom panel). Even after normalising the FACS data, we observed a strong decrease in connexin-45 expression and an increase in connexin-40 expression in differentiated SFS.1-NKX2-5KO cells compared to the same differentiation conditions in SFS.1WT cells.

**Figure 4:**
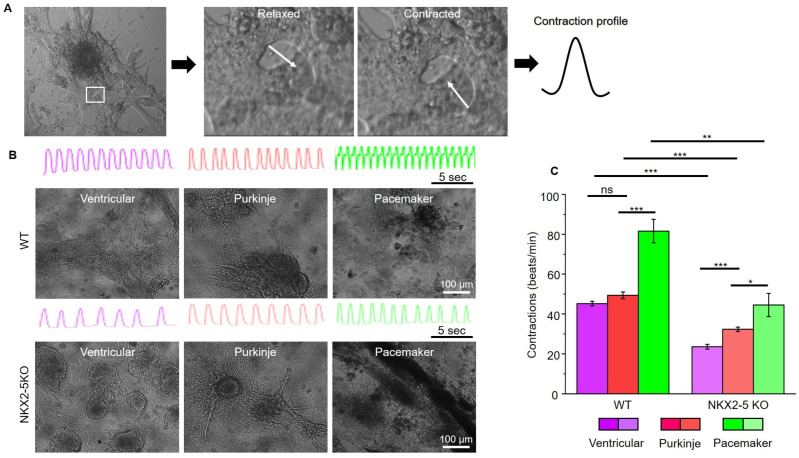
Contraction analysis of hiPSC-derived cardiac subtypes from SFS.1WT and SFS.1-NKX2-5KO. (**A**) Example of contraction profiling of beating hiPSC-derived cardiomyocytes. Within a beating tissue, a site with a high-contrast edge is selected; this edge is selected and analysed by its deflection using the musclemotion plugin for imageJ. Motion is indicated by the white arrow. (**B**) Exemplary pictures and contraction profiles (15 s) of SFS.1WT and SFS.1-NKX2-5KO, which were differentiated into cardiac subtypes. (**C**) Contraction analysis and comparison of cardiac subtypes differentiated from SFS.1WT and SFS.1-NKX2-5KO (* *p* < 0.05; ** *p* < 0.01; *** *p* < 0.001). ns means not significant.

After characterising the connexin expression pattern of SFS.1WT and SFS.1-NKX2-5KO, the contraction behaviour of the differentiated cells was investigated. For this purpose, videos of contracting cells were recorded and analysed for their contractile activity (Figure 4A). Ventricular-like cells differentiated from SFS.1WT showed an average contraction speed of 45.18 beats/min (±1.15 SEM), Purkinje-like cells showed a contraction speed of 49.33 beats/min (±1.70 SEM) and pacemaker-like cells showed a contraction speed of 81.60 beats/min (±5.92 SEM) (Figure 4B). SFS.1-NKX2-5KO-derived cardiac subtypes showed a contraction behaviour that was generally slowed compared to SFS.1WT-derived cells (Figure 4B,C). SFS.1-NKX2-5KO-derived ventricular cells contracted at a rate of 23.53 beats/min (±1.18 SEM), Purkinje-like cells at a rate of 32.29 beats/min (±1.11 SEM) and pacemaker-like cells at a rate of 44.48 beats/min (±5.82 SEM) (Figure 4C). Overall, knockout of NKX2-5 appears to have regulatory effects on connexin 45 expression and reduces contractility of hiPSC-derived cardiac subtypes.

**Figure 5:**
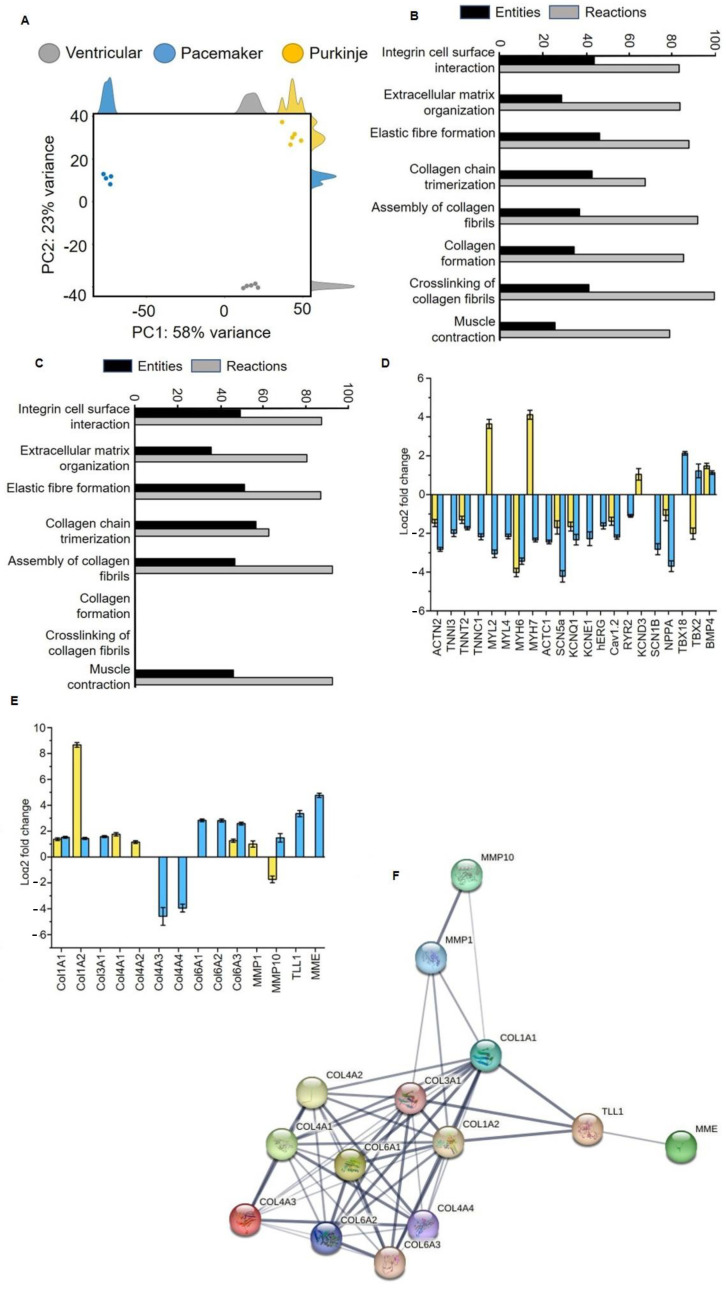
Transcriptomic analysis of hiPSC-derived cardiac subtypes from SFS.1WT and SFS.1-NKX2-5KO. (**A**) Principal component analysis of the transcriptomic data of SFS.1-NKX2-5KO differentiated Purkinje-like cells versus SFS.1WT differentiated ventricular-like and pacemaker-like cells. (**B**) Gene ontology analysis of the most highly regulated cellular signalling pathways in SFS.1-NKX2-5KO differentiated Purkinje-like cells versus SFS.1WT differentiated ventricular-like cells. (**C**) Gene ontology analysis of the most highly regulated cellular signalling pathways in SFS.1WT differentiated pacemaker-like cells versus SFS.1WT differentiated ventricular-like cells. (**D**) Transcriptomic alteration of myocardial genes in Purkinje-like cells (yellow) and pacemaker-like cells (blue) compared to differentiated ventricular-like cells. All genes shown have a *p*-value < 0.001 if changed in expression. (**E**) Transcriptomic alteration of collagen-network forming genes in Purkinje-like cells (yellow) and pacemaker-like cells (blue) compared to differentiated ventricular-like cells. All genes shown have a *p*-value < 0.001 if changed in expression. (**F**) Schematic representation of the interaction network of the altered collagen network forming proteins that were altered in the transcriptomic data shown in (**E**). Graphic was created with String-db.org.

Analysis of the transcriptome of differentiated SFS.1WT and NKX2-5KO cells showed clear distinctness of the three cardiac subtypes: ventricular-like and pacemaker-like (differentiated from SFS.1WT) and Purkinje-like (differentiated from SFS.1-NKX2-5KO). Principal component analysis (PCA) shows that samples of the same differentiation are very similar at the transcriptome level (about 23% variance within a group), but there is a clear expression difference between differentiated SFS.1WT (ventricular and pacemaker-like) and NKX2-5KO (Purkinje-like) cells. The transcriptomic difference between the differentiated cell types was 58% (Figure 5A). Gene ontology analysis was performed to group the expressed genes according to their function. Interestingly, the major groups altered in the transcriptome were related to extracellular matrix (ECM) formation and muscle contraction. Specifically, GO analysis of Purkinje-like cells compared to ventricular-like cells revealed the following major pathways altered in the transcriptome: Integrin-cell surface interaction (44.19% entities, 83.00% responses), organisation of extracellular matrix (28.90% entities, 84.01% responses), formation of elastic fibres (46.67% entities, 88.23% responses), trimerisation of collagen chains (43.18% entities, 67.86% reactions), collagen fibril assembly (37.31% entities, 92.31% reactions), collagen formation (34.78% entities, 85.78% reactions), collagen fibril cross-linking (41.67% entities, 100% reactions), muscle contraction (25.86% entities, 79.25% reactions) (Figure 5B). Go analysis was performed in the same way with transcriptomic data comparing pacemaker-like cells and ventricular-like cells differentiated from SFS.1WT. By analysing the same signalling pathways here, we can show differences between differentiated Purkinje-like cells and pacemaker-like cells. The previously mentioned signalling pathways were altered in pacemaker-like cells in the following ways: Integrin–cell surface interaction (53.49% entities, 94.55% responses), extracellular matrix organisation (38.72% entities, 87.15% responses), elastic fibre formation (55.56% entities, 94.12% responses), collagen chain trimerisation (61.36% entities, 67.86% reactions), collagen fibril assembly (50.75% entities, 100% reactions), collagen formation (0% entities, 0% reactions), collagen fibril cross-linking (0% entities, 0% reactions), muscle contraction (50% entities, 100% reactions) (Figure 5C).

The transcriptomic data were analysed in more detail for changes in cardiac genes and genes involved in cardiac collagen formation and polymerisation. Analysis of myocardial genes focused on the expression of sarcomeric genes and ion channels. Typical sarcomeric genes such as ACTN2, TNNI3, TNNT2, TNNC1, MYL2, MYL4, MYH6, MYH7 and ACTC1 were generally downregulated in pacemaker-like cells. In Purkinje-like cells, TNNI3, TNNC1, MYL4 and ACTC1 were unchanged in expression and MYL2 and MYH7 were upregulated. ACTN2, TNNT2 and MYH6 were downregulated. The exact expression levels can be found in Table 1.

The expression of myocardial ion channels showed a similar pattern to that of sarcomeric genes. Pacemaker-like cells showed a general downregulation of the myocardial ion channels SCN5a, KCNQ1, KCNE1, hERG, Cav1.2, RYR2 and SCN1B. Only the expression of KCND3 was unchanged. Purkinje-like cells showed downregulation for SCN5a, KCNQ1 and Cav1.2 and upregulation for KCND3. The expressions of KCNE1, hERG RYR2 and SCN1B were unchanged (Table 1). Pacemaker-like cells showed upregulation for the sinus node genes TBX18 and TBX2 and for the cardiac development gene BMP4. Purkinje-like cells showed downregulation for TBX2 and unchanged expression of TBX18. BMP4 was found to be upregulated (Table 1, Figure 5D).

HiPSC-derived pacemaker-like cells and Purkinje-like cells also differed greatly in the expression of collagen-forming and collagen-degrading genes. The analysis of these genes can be found in Figure 5E and Table 2. The Purkinje-like cells showed upregulated expression of collagen I type A1 and high A2 (Col1A1, Col1A2), collagen IV type A1 and A2 (Col4A1, Col4A2) and collagen VI type A3 (Col6A3). The expression of collagen III type A1 (Col3A1), collagen IV type A3 and A4 (Col4A3, Col4A4), collagen VI type A1 and A2 (Col6A1, Col6A2) was unchanged. Pacemaker-like cells showed increased expression of collagen I type A1 and A2 (Col1A1, Col1A2), collagen III type A1 (Col3A1) and collagen VI type A1, A2 and A3 (Col6A1, Col6A2, Col6A3). The expression of collagen IV type A1 and A2 (Col4A1, Col4A2) remained unchanged, whereas the expression of collagen IV type A3 and A4 (Col4A3, Col4A4) was strongly downregulated. Strong differences were also observed in the expression of the collagen-degrading enzymes matrix metallopeptidase 1 (MMP1), matrix metallopeptidase 10 (MMP10) and the collagen-cleaving/maturing enzymes membrane metalloendopeptidase (MME) and Tolloid-like 1 (TLL1). Purkinje-like cells showed upregulation of MMP1 and downregulation of MMP10, while the expression of TLL1 and MME was unchanged. In contrast, MMP1 expression was unchanged in pacemaker-like cells, while MMP10 expression was increased. The expression of TLL1 and MME was also strongly increased in pacemaker-like cells. In conclusion, the physiological and transcriptomic data suggest that knockout of NX2-5 and application of a Purkinje cell differentiation protocol results in a new cardiac cell type in which expression and function of myocardial and nodal proteins are inhibited. There is also increased secretion and polymerisation of collagen, which separates the cells from the surrounding cells.

## 3. Materials and Methods

### 3.1. Cell Culture

Sendai-Foreskin 1 wild-type human-induced pluripotent stem cells (SFS.1WT) were cultured on 1:75 diluted Matrigel^®^-coated 6-well plates with daily media changes. Matrigel^®^ (Becton Dickinson #354263) dilution was performed with KO-DMEM (Life Technologies (Carlsbad, CA, USA) #10829018) supplemented with 5% KnockOut Serum Replacement (KSR, Thermo Fisher (Waltham, MA, USA) #10828028) and 1 × Penicillin/Streptomycin/Glutamine (PSG, Thermo Fisher (Waltham, MA, USA) #10378016). FTDA-medium served as the maintenance medium, which was composed of DMEM/F12 (Invitrogen (Waltham, MA, USA) #21331020), 1 × PSG (Life Technologies #10378016), 1% CD Lipid Concentrate (Invitrogen #11905031), 5 µg/mL ITS (Becton Dickinson (Franklin Lakes, NJ, USA) #354351), 0.1% human serum albumin (HSA, NeoLab (Heidelberg, Germany) #05-720-1B), 10 ng/mL FGF-2 (PeproTech (Neuilly-sur-Seine, France) #100-18B), 0.2 ng/mL TGF-β (Abcam (Cambridge, UK) #Ab50036), 50 nM Dorsomorphin (Santa Cruz (Dallas, TX, USA) #sc200689) and 5 ng/mL Activin A (Thermo Fischer #PHC9564). Every 4 days, the cells reached complete confluence and were then split. The cells were washed once with Phosphate Buffered Saline (PBS, Life Technologies #D8537-500ML) and detached with 1 mL Accutase solution (Sigma Aldrich (St. Gallen, Switzerland) #A6964) supplemented with 10 µM Y-27632 ROCK Inhibitor (Sigma Aldrich #1254/10). After 10–12 min at 37 °C, when the cells completely detached, 1 mL FTDA + 10 µM Y-27632 ROCK Inhibitor was added to the cells and the cell suspension was transferred to a 15 mL Falcon tube. The cells were pelleted via centrifugation for 3 min at 200× *g*. The supernatant was removed and the cells were resuspended in 5 mL FTDA + 10 µM Y-27632 ROCK Inhibitor. About 650,000 cells were re-seeded onto new Matrigel^®^-coated 6-well plates.

### 3.2. NKX2-5 Knockout Generated with CrispR-Cas9

Wild-type SFS.1-hiPSCs (SFS.1WT) were seeded onto 1:75 diluted Matrigel^®^-coated 6-well plates at a rate of 150,000 cells/well. After 24 h, the cells were treated with the CrispR-Cas9 NKX2-5 Knockout Kit (Origene (Rockville, MD, USA) #KN511024) as described in the manufacturers’ instructions. The CrispR-Cas9 NKX2-5 Knockout Kit contained an EF1a-Luci-P2A-Puro oligo for co-transfection with the CrispR-Cas9 plasmid. This enabled positive cell selection for CrispR-Cas9-modulated cells via Puromycin selection. Twenty-four hours after transfection, Puromycin selection, at a concentration of 5 μg/mL, was undertaken for 14 days. Afterwards, the remaining cells were passaged onto 1:75 diluted Matrigel^®^-coated 6-well plates at a density of 100,000 cells/well so that colonies of single-cell origin could form. After 17 days, cell colonies had formed, and clones were picked for further cultivation. The new clones were named SFS.1-NKX2-5-KO.

### 3.3. Differentiation of Cardiac Subtypes

Cells of the cell lines SFS.1WT or SFS.1-NKX2-5KO were seeded onto 1:400 diluted Matrigel^®^-coated 24-well plates at a density of 500,000 cells/well in Day 0 differentiation medium. Day 0 differentiation medium consisted of KO-DMEM supplemented with 1 × ITS, 10 μM Y-27632, 1 × PSG, 5 ng/mL Activin A (Thermo Fisher #PHC9564), 10 ng/mL FGF-2 (Peprotech #100-18B), 0.5–1 ng/mL BMP4 (R&D (McKinley Place, NE, USA) #314-BP-050), and 1 μM CHIR99021 (Tocris (Bristol, UK) #4423/10). After 24 h in culture, medium was changed to TS-ASC medium consisting of KO-DMEM supplemented with 1 × PSG, 1 × Transferrin/Selenium (TS) Solution, 250 μM 2-phospho-ascorbate (Sigma-Aldrich # 49752). A 100 × TS stock solution was prepared by solving 55 mg human transferrin (Sigma Aldrich #T8158-100MG) and 0.067 mg Selenite (Sigma Aldrich #S5261-10G) in 100 mL PBS. The cells were treated daily with 2 mL/well of TS-ASC medium. On differentiation days 2 and 3, the WNT-inhibitor C59 (Tocris #5148/10) was added at a final concentration of 0.2 mM. Differentiation continued until day 8, when the cells started autonomously beating [6,24].

### 3.4. Ventricular-like Differentiation

After 8 days of differentiation, the young, beating cardiomyocytes were transferred onto 1:400 diluted Matrigel^®^-coated 24-well plates with additional 0.1% Gelatine coating (Sigma Aldrich #G1393-20ML) for 1 h at RT. One well of young cardiomyocytes was split in a ratio of 1:4 into new wells. The cells were split and cultivated in KO-THAI medium consisting of KO-DMEM supplemented with 1 × PSG, 1 × ITS, 0.2% HSA, 250 μM phospho-ascorbate, 0.008% Thioglycerol (Sigma Aldrich #M1753). Splitting was performed in KO-THAI medium + 10 μM Y-27632. The cells were cultivated in KO-THAI for 28 days until a more mature ventricular-like phenotype was achieved [6,24].

### 3.5. Pacemaker-like Differentiation

After 8 days of differentiation, the young, beating cardiomyocytes were transferred onto 1:400 diluted Matrigel^®^-coated 24-well plates with additional 0.1% Gelatine coating for 1 h at RT. One well of young cardiomyocytes was split in a ratio of 1:4 into new wells. Splitting was performed with KO-THAI medium + 10 μM Y-27632. Twenty-four hours after splitting, the medium was changed to pacemaker differentiation medium consisting of KO-DMEM supplemented with 1 × PSG, 1 mM CaCl_2_ and 10% FBS. The cells were cultivated for 28 days in pacemaker differentiation medium until a pacemaker-like phenotype was achieved [13,14].

### 3.6. Conduction System-like Differentiation

After 8 days of differentiation, the young, beating cardiomyocytes were transferred onto 1:400 diluted Matrigel^®^-coated 24-well plates with additional coating with 0.1% Gelatine for 1 h at RT. One well of young cardiomyocytes was split in a ratio of 1:4 into new wells. Splitting was performed with KO-THAI medium + 10 μM Y-27632. Twenty-four hours after splitting, the medium was changed to conduction system differentiation medium consisting of KO-THAI medium supplemented with 10 µM Forskolin (STEMCELL Technologies (Köln, Germany) #72114). The cells were cultivated for 28 days in conduction system differentiation medium until a conduction system-like phenotype was achieved [16,17].

### 3.7. Polymerase Chain Reaction

Young hiPSC-derived cardiomyocytes at day 4 of differentiation were detached from the well surface by digestion with 0.5 mL 1 × TrypLE Select (Life Technologies #12563011) + 10 μM Y-27632 for 12 min. The digestion was aborted with 0.5 mL TS-ASC medium + 10 μM Y-27632. The cells were transferred to 1.5 mL Eppendorf tubes and centrifuged for 3 min at 200× *g*. The resulting cell pellet was separated from the supernatant and used for RNA isolation. RNA isolation was performed with the NucleoSpin RNA XS Kit (MACHEREY-NAGEL GmbH & Co. KG (Düren, Germany) #740902.50) according to the manufacturer instructions. RNA concentration and quality was controlled by photospectrometry. Subsequent cDNA synthesis was performed with 1 mg of isolated RNA using the High-Capacity cDNA Reverse Transcription kit (Thermo Fisher Scientific # 4368814) according to the manufacturer’s instructions. For NKX2-5-cDNA synthesis from RNA, the following primers were used: forward, AAGTGTGCGTCTGCCTTTCCCG; reverse, TTGTCCGCCTCTGTCTTCTCCA; expected cDNA fragment size, 146 bp. The NKX2-5 fragment synthesis was verified by electrophoresis in a 3% agarose gel.

### 3.8. FACS Sorting

Maturated hiPSC-derived cardiac myocytes with either ventricular-, pacemaker- or conduction system-like specification were detached at day 35 of maturation with 0.5 mL 10 × TrypLE (Thermo Fisher (Waltham, MA, USA)) + 10 μM Y-27632 for 12 min. The digestion was stopped by addition 0.5 mL of the corresponding maturation medium + 10 μM Y-27632. The cells were transferred to a 1.5 mL Eppendorf tube and centrifuged for 3 min at 200× *g*. the supernatant was removed and the pellet was washed two times with PBS. Then, the pellets were fixed for 10 min with a 3.7% PFA solution. The fixed pellets were washed twice with PBS. The washed pellets were blocked for 1 h with a 0.5% BSA solution. Afterwards, the pellets were washed twice with PBS. The pellets were stained with the following, conjugated antibodies against connexin 43, connexin 40 and connexin 45 (Santa Cruz #sc-271837 AF647; #sc-365107 AF594; #sc-374354 AF488). The respective antibody was used 1:500 in a 0.1% BSA solution for 2 h at RT. The stained pellets were washed twice with PBS and then FACS-analysed.

### 3.9. Immunofluorescence Staining

Glass coverslips 12 mm in length were coated with 1:400 Matrigel^®^, and directly before seeding, the cells were additionally incubated with 0.1% gelatine solution (Sigma Aldrich #G1393) for 30 min at room temperature. For detaching, cells were incubated with 10× TrypLE supplemented with 10 µM Y-27632 ROCK Inhibitor for 5–10 min. Cells were seeded in a density of 30,000 cells per coverslip, and subsequently cultured at 37 °C and 5% CO_2_ for 2 more days with medium exchange daily. When fully adherent and spread, cells were fixed with 3.7% PFA (Paraformaldehyde, Sigma Aldrich #158127) for 30 min, treated with 0.2% Triton-X (Roth #3051) for 10 min and blocked with 0.5% BSA solution (Bovine Serum Albumin, Sigma Aldrich #A9418) for 60 min. Cells were washed with PBS and incubated for 1 h with antibodies for connexin 43, connexin 40 and connexin 45, dissolved 1:400 in 0.5% BSA solution. Subsequently, cells were washed with PBS supplemented with DAPI (Thermo Fisher #62247) 1:1000 and mounted on cover slides with AquaPolymount (Polysciences, Inc. (Warrington, PA, USA) #18696). Sample imaging was performed with a confocal microscope (Leica DMI 4000 B) at 63× magnification.

### 3.10. Contraction Analysis

hiPSC-derived cardiomyocytes were detached as described above and seeded into 1:400 diluted Matrigel^®^ and 0.1% gelatine solution-coated 24-well plates. Cells were cultivated at 37 °C and 5% CO_2_ with medium change every 48 h. For imaging the cells, a Hamamatsu C11440 camera and the Fiji plugin micro manager were used. The cells were imaged for 30 s at 30 Hz sampling rate and 10× magnification under β-adrenergic stimulation with 10 µM isoprenaline at room temperature. The contraction frequencies were determined, and contraction profiles were made with the musclemotion macro for Fiji.

### 3.11. Transcriptomic Analysis

Maturated hiPSC-derived cardiac myocytes with either ventricular-, pacemaker- or conduction system-like specification were detached at day 35 of maturation with 0.5 mL 10 × TrypLE Thermo Fisher (Waltham, MA, USA) + 10 μM Y-27632 for 12 min. The TrypLE reaction was stopped by the addition of 0.5 mL maturation medium + 10 μM Y-27632. The cells were centrifuged for 3 min at 200× *g*. The resulting supernatant was removed and the cell pellet washed in 0.5 mL PBS. The cells were centrifuged once more for 3 min at 300× *g*. The cell pellet was separated from the supernatant. Whole RNA-Isolation was performed with the NucleoSpin RNA, Mini kit for RNA purification (Macherey-Nagel (Düren, Germany) #740955.50). Next-generation sequencing (NGS) was performed by the Core Facility Genomics of the Medical Faculty of the WWU Münster, University using the NextSeq2000 (Illumina, Inc. (San Diego, CA, USA) #20038897). Transcriptomic data analysis was performed by the Institute of Medical Informatics, University of Münster, Münster, Germany. Quantification of the RNAseq data was performed using Salmon. Subsequent analyses were conducted using R 4.3.1, determining differentially expressed genes using the R package DESeq2 [25,26].

## 4. Discussion

Knockout of the NKX2-5 gene in SFS.1WT-hiPSC was performed and a NKX2-5-deficient cell line, SFS.1-NKX2-5KO, was generated (Figure 2A–C). Cardiac subtype-specific differentiation protocols were applied to SFS.1WT and SFS.1-NKX2-5KO, and the resulting cells were analysed for their connexin expression patterns and contractile behaviour. Since it is difficult to unambiguously distinguish cells of different cardiac subtypes based on specific morphological or genetic markers, we focused on the parameters known to be unique to each cardiac subtype. Despite major inconsistencies in the data characterising cardiac subtypes, experts agree that the expression patterns of connexins in different cardiac tissues are unique. This feature may make sense, because physiological cardiac function is mediated by specific signal conduction through the different cardiac tissues. This well-timed conduction is mediated via specific connexins in the gap junctions of specific cardiac tissues, resulting in variable connectivity properties between cells, and thus adaptive signal processing speed. Here, cardiac connexin 43 forms channels with a conductance of 120 pS and V_1/2_ = 60 mV, connexin 40 forms channels with a conductance of 150 pS and V_1/2_ = 40 mV, and connexin 45 shows a conductance of 30 pS and V_1/2_ = 13 mV. [27]. The conduction properties of connexin 45 indicate a high voltage sensitivity, as needed for fast signal propagation, as it is indispensable for correct SAN function where connexin 45 is highly expressed [28]. The second most voltage-sensitive connexin is connexin 40, which is highly expressed in the cardiac conduction system [29,30,31]. Connexin 43 is the least voltage-sensitive connexin studied here and is predominantly expressed in the working myocardium, where a moderate conduction velocity is appropriate. Comparison of the connexin expression profiles of SFS.1WT- and SFS.1-NKX2-5KO-derived cardiac subtypes revealed a high proportion of connexin 43-expressing chamber-like cells and a high proportion of connexin 45-expressing pacemaker-like cells in SFS.1WT-derived cells. Even with the Purkinje cell differentiation protocol, we could not detect increased connexin 40 expression in SFS.1WT cells. In contrast, we found highly connexin 40-expressing Purkinje-like cell populations derived from SFS.1-NKX2-5KO treated with one of the three cardiac subtype differentiation protocols. Among these, the Purkinje cell differentiation protocol resulted in the highest connexin 40 expression. Connexin 45 expression was greatly reduced in SFS.1-NKX2-5KO derived cardiac cells (Figure 3A–C). Contractility was significantly reduced in SFS.1-NKX2-5KO-derived cardiac cells. Both comparison of equal cardiac subtypes differentiated from SFS.1WT and SFS.1-NKX2-5KO and comparison of total contractility between different cardiac subtypes derived from the same hiPSC lineage showed a reduction in contraction in SFS.1-NKX2-5KO-derived cells (Figure 3A,B). This may be consistent with the known function of NKX2-5 as an expression activator for genes critical for the proper function of the working myocardium [32,33]. Since the expression of cardiac muscle genes might be reduced by the NKX2-5 knockout, a decreased contractility of the differentiated cardiac cells from SFS.1-NKX2-5KO is to be expected, as seen in Figure 3. In addition, abolition of the fast-conducting connexin 45 may lead to changes in the physiological behaviour of SFS.1-NKX2-5KO-derived cells during the maturation period of 35 days. Since connexin 45-deficient tissue may be less conductive, slower signal transduction and a potential decrease in tissue contractility over time are a logical consequence. Together with the increased expression of connexin 40 and the attenuated expression of connexin 45 in SFS.1-NKX2-5KO-derived cardiac cells, regardless of the differentiation protocol used, we suggest that the differentiation approach presented may provide evidence that the resulting cells more closely resemble a CSLC phenotype.

The Purkinje-like cells can be clearly distinguished genetically and functionally from the differentiated pacemaker-like cells and ventricular cells, as shown by PCA (Figure 5A). GO analysis of the most affected signalling pathways of the differentiated Purkinje-like cells shows that these cells differ from the ventricular-like cells and the pacemaker-like cells, especially with regard to collagen formation and cross-linking of collagen fibrils (Figure 5B,C). Furthermore, detailed analysis of the transcriptome data of the differentiated Purkinje-like cells clearly shows downregulation of sarcomere genes and myocardial ion channels, demonstrating that the resulting cells do not resemble a myocardial phenotype and are also distinct from the pacemaker-like cells analysed in this study (Figure 5D, Table 1).

**Figure 6:**
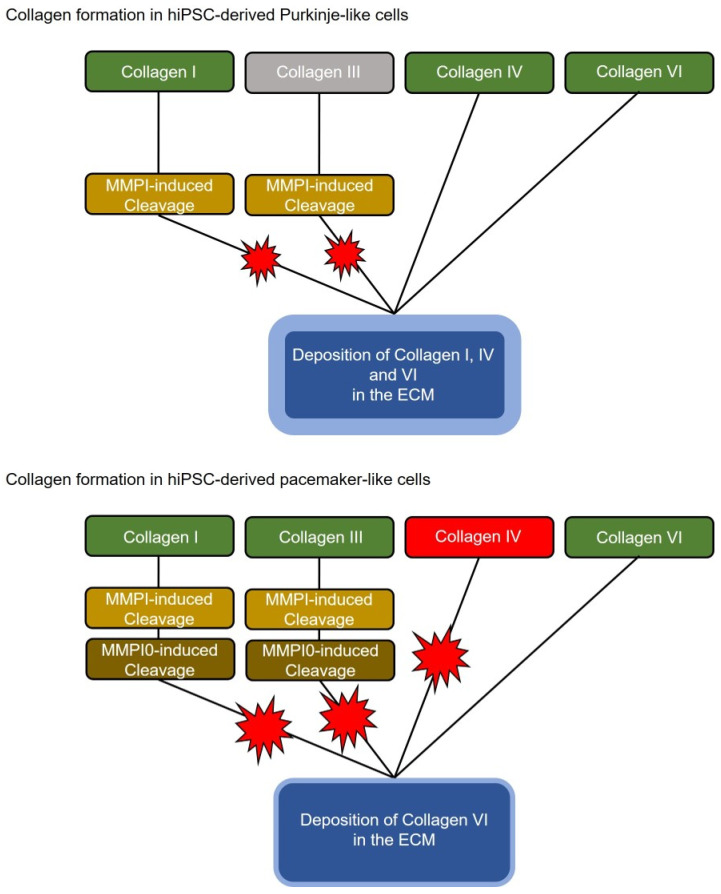
Schematic representation of altered collagen deposition in hiPSC-derived Purkine-like cells and pacemaker-like cells for the generation of an extracellular matrix.

Interestingly, analysis of the transcriptome data showed that the Purkinje-like cells have strong upregulation of the Col1A1 and especially the Col1A2 gene, while the collagen-degrading genes are not upregulated on average, with of MMP1 and downregulation of MMP10. This should result in an increased collagen secretion and extracellular accumulation in the Purkinje-like cell culture (Figure 6, top). Increased collagen formation is a typical feature of the cardiac conduction system and Purkinje fibres in particular. This effect is unlikely or attenuated in the differentiated pacemaker-like cells, as the Col1A1, Col1A2, Col3A1, Col6A1, Col6A2 and Col6A3 genes are each slightly upregulated, but genes with collagen-degrading activity, MMP10 and MME, are also strongly upregulated (Figure 5E, Table 2). Therefore, strongly increased collagen secretion in pacemaker-like cells is not necessarily to be expected (Figure 6, bottom). Matrix metalloproteinase 1 (MMP1) and matrix metalloproteinase 10 (MMP10) are known to cleave and thus degrade collagen. MMP1 mainly cleaves collagens I, II and III and MMP10 mainly cleaves collagen III (Figure 5F). Since collagen-degrading genes are not upregulated in Purkinje-like cells, this should result in increased accumulation of collagen IV and VI in the ECM. Interestingly, pacemaker-like cells show increased expression of TLL1 and MME. TLL1 leads to increased polymerisation of collagen, while MME is an active interaction partner for TLL1. TLL1 cleavage is induced by MME, which could be a self-regulatory process triggered by the pacemaker-like cell to regulate proper collagen polymerisation and avoid overactivity of TLL1 (Figure 5F). However, whether this is really the case needs to be clarified in detail in further studies.

With regard to differentiation to CSLC, the accumulation of collagen in the ECM is reasonable. Collagen acts like an electrical insulator in the ECM and prevents current transmission from cell to cell [34,35]. In our cell system presented here, this means that we obtain electrically coupled cell strands that are electrically isolated from the surrounding tissue. This creates electrically insulated biological cables that conduct generated potentials from one point to the next without increasing activation of the surrounding cells, which corresponds to the function of the conduction system in the human heart [36,37]. The differentiation of hiPSC into adult and fully functional CSLC of the Purkinje-like cell types still needs to be optimised greatly, but with the approach presented here, we have taken a step forward in generating functional cardiac CSLC and provided the scientific community with a new tool to generate electrically conductive biological cables with cardiac properties for use in tissue engineering and disease modelling.

## 5. Conclusions

CrispR-Cas9-driven knockout of NKX2-5 in SFS.1WT-hiPSC resulted in a change in connexin expression pattern and contraction behaviour after cardiac differentiation. The loss of connexin 45 expression and the increased expression of connexin 40 and connexin 43 compared to SFS.1WT cells indicated the formation of a specialised cardiac cell type. Additional maximal activity analysis revealed reduced contractility of SFS.1-NKX2-5KO-derived cardiac cells, supported by reduced expression of contractile proteins, myocardial ion channels and induced collagen secretion, indicating enhanced differentiation to CSLC. The CSLCs generated are now available to the scientific community and can be used to further characterise disease processes in the human cardiac conduction system, which are otherwise difficult to study. Furthermore, the CSLCs can be used for tissue engineering. For example, it would be conceivable to connect hiPSC-derived ventricular or atrial cells with the generated CSLCs in order to investigate the signal transmission between different cardiac tissues, and thus also to model diseases of the human cardiac conduction system. The use of CSLCs as biological, isolated cables with a defined rate of stimulus transmission would also be a possible application to specifically stimulate cells or cell clusters in the petri dish, and thus create controlled experimental conditions for physiological experiments.

## Figures and Tables

**Table 1:** Expression levels of sarcomeric genes and myocardial ion channels and cardiac developmental genes in hiPSC-derived pacemaker-like and Purkinje-like cells. Red label shows downregulation, green label shows upregulation of a gene.

Purkinje-like				Pacemaker-like		
Gene Name	Log2-Fold Change	Standard Error	Significance	Log2-Fold Change	Standard Error	Significance
ACTN2	−1.46706	0.181589	1.13036 × 10^−17^	−2.8243	0.103478	5.8962 × 10^−166^
TNNI3	unchanged	-	-	−1.9942	0.175623	8.28654 × 10^−32^
TNNT2	−1.30222	0.184863	4.49837 × 10^−14^	−1.73748	0.085314	2.17479 × 10^−94^
TNNC1	unchanged	-	-	−2.1751	0.152601	3.17063 × 10^−48^
MYL2	3.644449	0.234498	2.01014 × 10^−56^	−3.05391	0.196635	8.84141 × 10^−57^
MYL4	unchanged	-	-	−2.16469	0.120022	6.61284 × 10^−75^
MYH6	−4.02142	0.218756	3.62909 × 10^−77^	−3.43693	0.162719	2.1178 × 10^−101^
MYH7	4.121104	0.230313	3.28128 × 10^−73^	−2.34301	0.094976	2.5606 × 10^−136^
ACTC1	unchanged	-	-	−2.44346	0.095251	3.9181 × 10^−147^
SCN5a	−1.69562	0.341282	2.20255 × 10^−8^	−4.21967	0.294333	7.48243 × 10^−49^
KCNQ1	−1.65104	0.233765	3.6577 × 10^−14^	−2.32783	0.274448	2.68076 × 10^−19^
KCNE1	unchanged	-	-	−2.27877	0.357455	3.19689 × 10^−12^
hERG	unchanged	-	-	−1.61788	0.157384	7.53886 × 10^−27^
Cav1.2	−1.37522	0.206129	6.35576 × 10^−13^	−2.19107	0.105635	7.37335 × 10^−98^
RYR2	unchanged	-	-	−1.09889	0.071976	7.80597 × 10^−55^
KCND3	1.042517	0.300723	2.55367 × 10^−5^	unchanged	-	-
SCN1B	unchanged	-	-	−2.82108	0.285001	3.80583 × 10^−25^
NPPA	−1.07016	0.284895	7.34624 × 10^−6^	−3.69601	0.278952	4.18312 × 10^−42^
TBX18	unchanged	-	-	2.126404	0.094746	6.115 × 10^−114^
TBX2	−2.01272	0.290398	9.04765 × 10^−14^	1.217084	0.352638	1.78033 × 10^−5^
BMP4	1.476836	0.137793	1.15351 × 10^−28^	1.134497	0.093217	2.67215 × 10^−36^

**Table 2:** Expression levels of cardiac collagen-forming and degrading genes in hiPSC-derived pacemaker-like cells and Purkinje-like cells. Red label shows downregulation, green label shows upregulation of a gene.

Purkinje-like				Pacemaker-like		
Gene Name	Log2-Fold Change	Standard Error	Significance	Log2-Fold Change	Standard Error	Significance
Col1A1	1.379072	0.108083	2.14 × 10^−39^	1.521192	0.079142	1.32 × 10^−84^
Col1A2	8.666613	0.18206	4.45 × 10^−5^	1.439349	0.080931	5.7 × 10^−73^
Col3A1	unchanged	-	-	1.57902	0.074156	6.3 × 10^−103^
Col4A1	1.758753	0.130923	5.47 × 10^−43^	unchanged	-	-
Col4A2	1.157226	0.109435	4.09 × 10^−28^	unchanged	-	-
Col4A3	unchanged	-	-	−4.58596	0.689022	4.19 × 10^−13^
Col4A4	unchanged	-	-	−3.94712	0.304542	1.47 × 10^−40^
Col6A1	unchanged	-	-	2.836245	0.100078	1.2 × 10^−178^
Col6A2	unchanged	-	-	2.820352	0.118623	6.7 × 10^−127^
Col6A3	1.26064	0.121888	9.22 × 10^−27^	2.584923	0.110618	6 × 10^−123^
MMP1	1.002105	0.239981	1.14 × 10^−6^	unchanged	-	-
MMP10	−1.72784	0.255748	3.37 × 10^−13^	1.480713	0.328658	1.6 × 10^−7^
TLL1	unchanged	-	-	3.360525	0.240076	1.38 × 10^−46^
MME	unchanged	-	-	4.768142	0.170294	2.2 × 10^−174^

## Data Availability

The datasets generated during and/or analysed during the current study are available from the corresponding author on reasonable request.
